# Differences in Age-Standardized Mortality Rates for Avoidable Deaths Based on Urbanization Levels in Taiwan, 1971–2008

**DOI:** 10.3390/ijerph110201776

**Published:** 2014-02-05

**Authors:** Brian K. Chen, Chun-Yuh Yang

**Affiliations:** 1 Department of Health Services Policy and Management, Arnold School of Public Health, University of South Carolina, 915 Greene Street, Columbia, SC 29208, USA; 2 Department of Public Health, College of Health Sciences, Kaohsiung Medical University, 100 Shih-Chuan 1st Road, Kaohsiung 807, Taiwan; E-Mail: chunyuh@kmu.edu.tw

**Keywords:** urbanization, avoidable mortality, health disparities, Taiwan, breast cancer, lung cancer, ischemic heart disease

## Abstract

The World is undergoing rapid urbanization, with 70% of the World population expected to live in urban areas by 2050. Nevertheless, nationally representative analysis of the health differences in the leading causes of avoidable mortality disaggregated by urbanization level is lacking. We undertake a study of temporal trends in mortality rates for deaths considered avoidable by the Concerted Action of the European Community on Avoidable Mortality for four different levels of urbanization in Taiwan between 1971 and 2008. We find that for virtually all causes of death, age-standardized mortality rates (ASMRs) were lower in more urbanized than less urbanized areas, either throughout the study period, or by the end of the period despite higher rates in urbanized areas initially. Only breast cancer had consistently higher AMSRs in more urbanized areas throughout the 38-year period. Further, only breast cancer, lung cancer, and ischemic heart disease witnessed an increase in ASMRs in one or more urbanization categories. More urbanized areas in Taiwan appear to enjoy better indicators of health outcomes in terms of mortality rates than less urbanized areas. Access to and the availability of rich healthcare resources in urban areas may have contributed to this positive result.

## 1. Introduction

The World is experiencing rapid urbanization, with significant implications for health. In 2010, the number of individuals living in urban areas crossed the threshold of 50% of the total World population for the first time in history [1]. It is estimated that by 2050, over 70% of the World population will be living in urban environments [2]. The continuing process of urbanization worldwide has engendered interest in both policy and academic circles, as its impact on health is often context-specific and difficult to generalize.

On the one hand, living in urban conglomerations intensifies exposures to adverse environmental [3], epidemiological [4,5], and social [6] factors that tend to impact health negatively, particularly for the urban poor. On the other, the greater wealth and concentration of healthcare services available in urban areas also mean that illnesses can be more effectively managed [7]. It is empirically unclear which of the two opposing forces on health dominates in any given context. As a result, it is of critical importance to understand whether the adverse health impact of urban residence can be overcome by one of its strongest benefits—the ready access to medical care—in a broad range of disease contexts.

Indeed, methodological and data challenges remain in the literature on place and health. First, existing literature often focuses on individual disease categories, and nationally representative data are scarce [1]. Differences in the socioeconomic, environmental, and epidemiological contributors to poor health may well emphasize differing aspects of the positive and adverse health factors of urban living, particularly when comparing across disease categories. Second, methodological shortcomings exist when definitions focus primarily on the physical rather than the socioeconomic context of urbanization [8]. Third, a simple urban-rural dichotomy may also mask health disparities along the urban-rural continuum [9].

To overcome these challenges, we study the implication of urbanization and health by using a multifactorial definition of urbanization that encompasses both socioeconomic and physical attributes of urban development, as well as data on all deaths in Taiwan from 1971 to 2008. Our primary objective is to trace the temporal trends in mortality from diseases considered to be amenable to public health or medical interventions in Taiwan, disaggregated by four levels of urbanization. In so doing, we aim to investigate whether the mortality benefits of urban residence are overall greater than their social and environmental harms in an export-driven economy such as that of Taiwan.

Our work contributes to the literature in two specific ways. First, there is very limited empirical literature on the impact of urbanization on health in Taiwan. Existing research often focuses on individual disease categories, uses survey samples or small, potentially non-nationally representative samples of death, or considers urbanization as incidental, rather than the primary covariate of interest. A 1998 study finds that more urbanized areas in Taiwan had increasing trends in certain cancer mortalities [10]. Other studies link urban environmental factors such as pollution, fluoridation, water hardness and arsenic contamination with coronary or cancer moralities [11,12,13,14,15,16,17]. Yet another study finds higher mortality rates from traffic accidents in rural areas of Taiwan between 1981 and 1990 [18]. The remainder of the literature on health and place in Taiwan focuses on prevalence or incidence of [19,20], rather than mortality from disease, differences in health care utilization patterns [21,22,23,24], or differences in risk factors [25] based on the urbanization level of the study population’s residence.

Second, our work contributes to the literature by providing important implications for Taiwanese policymakers in identifying the geographic distribution of different types of mortality. Such knowledge can contribute to a more efficient allocation of scarce healthcare resources depending on the prevalence of different causes of death between rural and urban areas in Taiwan, and may shed further light on the urban-rural health outcome divide in other Asian nations following a similar path of export-driven growth and development.

## 2. Experimental Section

### 2.1. Causes of Mortality

Our primary data source is the Taiwan National Death Certification Registry from 1971 to 2008. The registry includes variables such as gender, year of birth, and the date of and cause of death of all citizens. Cause of death coding, or the assignment of an International Classification of Diseases, 9th Revision, Clinical Modification (ICD-9-CM) code for the primary cause of death for each decedent, is considered very accurate in Taiwan because a death certificate must be issued by trained medical staff at Taiwan’s Household Registry Office within 30 days after a resident’s death [26]. 

From the recorded cause of death, we categorized “avoidable deaths” and “deaths due to other causes” using the definition established by the Concerted Action of the European Community on Avoidable Mortality (CAEC) [27]. CAEC classified deaths based on whether they are amenable to public health or medical interventions. For example, deaths from ischemic heart disease (IHD) can be prevented or delayed though public health interventions such as anti-tobacco programs, and through medical care such as surgery or prescription medications. Deaths from lung cancer are principally amenable to public health interventions through smoking cessation programs.

Because of the long-study period, the ICD-9-CM codes recorded in the death registry as the causes of death have been modified several times. However, the Taiwan National Death Registry recodes all previously available mortality data to reflect the latest version of the ICD-9-CM, so that the length of the study period should not cause concern with respect to coding consistency. While more updated classifications of amenable deaths are available, we selected the 1997 CAEC list because of its temporal proximity to the midpoint of our study period. We restrict our analyses to deaths at age 65 or before, again because of the long study period and significant changes in life expectancies over the study period.

### 2.2. Urbanization

We categorized all areas in Taiwan into four levels of urbanization based on the work by Tzeng and Wu [28], who created an eight-level composite score using key socioeconomic variables such as age structure, population density, employment rate, manufacturing density, mean male and female immigration rates, index of economic activities, mean annual per capita income and expenditures, mean daily amount of garbage per 1,000, mean number of telephones per family, educational level, number of physicians per 1,000, and availability of healthcare facilities in each jurisdiction. We combined levels 1–2, 3–4, 5–6, and 7–8 into four separate levels of urbanization, with 1 representing the most, and 4 the least urbanized jurisdictions in Taiwan.

Despite the age of this classification method, it remains the urbanization stratification method of choice by many researchers in Taiwan [29]. In fact, a study in 2006 using updated sociodemographic data from Taiwan’s towns and cities resulted in a classification of urbanization substantially similar to that of Tzeng-Wu [30].

### 2.3. ASMR

We age-standardized mortality rates in five-year intervals (1971 to 2005), except the last three years (2006–2008). We disaggregated the analysis by gender because some medical conditions are gender-specific, and others have traditionally been demonstrated to have gender-based differences in outcomes. We use the direct method to calculate age-standardized mortality rates (ASMRs) per 100,000 [31], as follows:

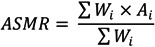

where *W*_i_ is the population in the *i*th age class of the reference population (the world population in 2000) and A_i_ is the age-specific mortality rate in the *i*th age class in Taiwan.

**Figure 1:**
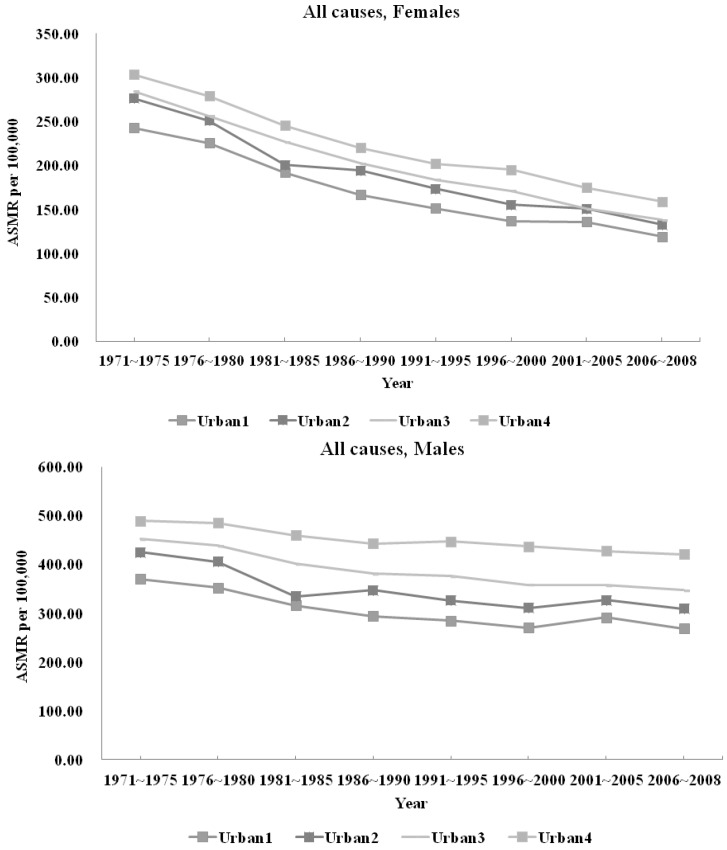
All cause mortality by urbanization level (Top: females; bottom: males).

**Figure 2:**
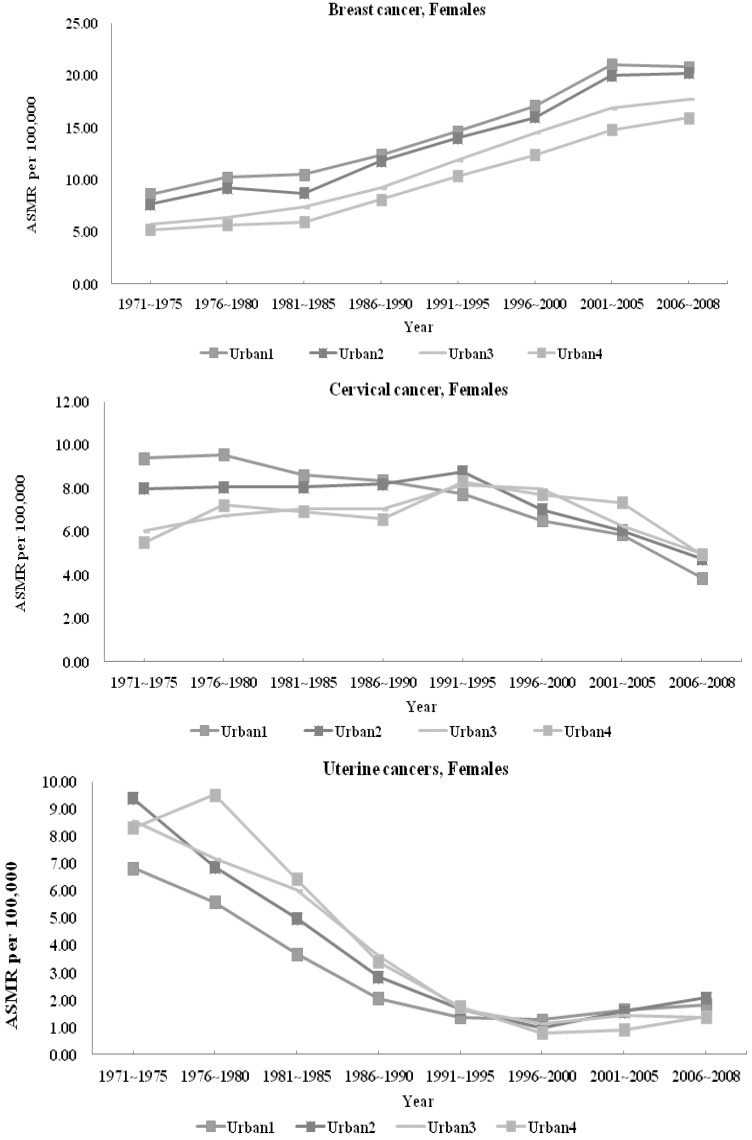
Avoidable Mortality by Urbanization Level, Gender-Specific Cancers.

**Figure 3:**
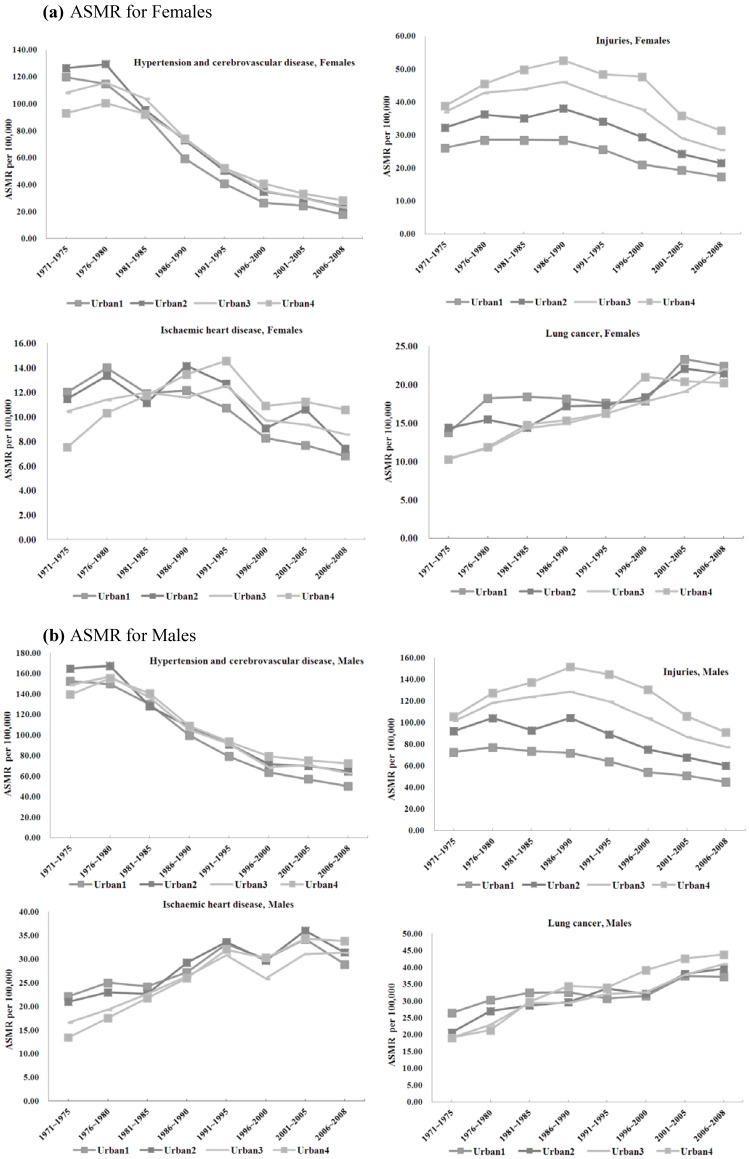
Avoidable Mortality by Urbanization Rates: Injuries, Hypertension, IHD, Lung Cancer (a: females, b: males).

The ASMR for each cause of avoidable mortality thus calculated is presented for all diseases in Table 1 (women) and Table 2 (men). Because of the large number of causes of death studied, for ease of presentation, we provide in Figure 1, Figure 2 and Figure 3 the line plots of only a subset of causes for which important changes in trends have been noted. We plot the temporal trends for all-cause mortality (Figure 1), breast, cervical, and uterine cancers (Figure 2), as well as injuries, hypertension, ischemic heart disease, and lung cancer (Figure 3a (women) and Figure 3b (men)). All values are presented without confidence intervals because our data include the entire population of all deaths in Taiwan.

**Table 1:** Standardized mortality ratio (SMR per 100,000 people), females.

Urban1								
Cause	Year							
	1971–1975	1976–1980	1981–1985	1986–1990	1991–1995	1996–2000	2001–2005	2006–2008
All causes	242.62	225.58	192.11	166.78	151.34	137.03	135.93	119.15
Appendicitis	0.17	0.15	0.06	0.04	0.02	0.01	0.04	0.00
Asthma	0.85	0.65	0.39	0.46	0.29	0.29	0.28	0.17
Breast cancer	8.61	10.20	10.52	12.37	14.65	17.07	21.02	20.79
Cervical cancer	9.38	9.56	8.61	8.35	7.73	6.52	5.86	3.87
Gallbladder disease	0.81	0.68	0.28	0.19	0.17	0.08	0.16	0.17
Hernia	0.00	0.01	0.06	0.01	0.00	0.01	0.00	0.00
Hodgkin’s disease	0.13	0.11	0.08	0.08	0.07	0.03	0.05	0.01
Hypertension/CVD *	119.68	114.52	91.96	59.26	40.79	26.56	24.32	17.94
Injuries	26.06	28.47	28.45	28.37	25.61	21.01	19.32	17.26
Ischemic heart disease	12.01	14.00	11.91	12.16	10.72	8.26	7.69	6.80
Lung cancer	13.74	18.20	18.44	18.14	17.61	17.87	23.31	22.38
Maternal mortality	1.27	0.78	0.44	0.26	0.19	0.19	0.13	0.09
Tuberculosis	10.29	5.34	3.08	1.60	0.95	0.76	0.44	0.23
Ulcers	2.68	1.52	1.12	0.84	0.54	0.64	0.69	0.34
Uterine cancer	6.83	5.58	3.68	2.06	1.37	1.26	1.64	1.82
**Urban2**								
**Cause**	**Year**							
	**1971–1975**	**1976–1980**	**1981–1985**	**1986–1990**	**1991–1995**	**1996–2000**	**2001–2005**	**2006–2008**
All causes	276.49	250.86	200.89	194.18	173.57	155.20	150.91	132.79
Appendicitis	0.07	0.14	0.08	0.03	0.01	0.02	0.01	0.03
Asthma	0.91	0.66	0.62	0.46	0.39	0.39	0.21	0.24
Breast cancer	7.65	9.20	8.70	11.79	14.00	15.97	19.99	20.19
Cervical cancer	7.99	8.06	8.05	8.20	8.77	7.02	6.06	4.74
Gallbladder disease	0.53	0.78	0.41	0.18	0.24	0.09	0.20	0.14
Hernia	0.03	0.02	0.02	0.01	0.00	0.00	0.01	0.00
Hodgkin’s disease	0.13	0.12	0.13	0.10	0.04	0.08	0.04	0.05
Hypertension/CVD *	126.46	129.27	95.47	72.74	50.21	34.74	30.14	23.74
Injuries	32.19	36.14	35.04	38.03	34.04	29.31	24.23	21.45
Ischemic heart disease	11.46	13.35	11.12	14.15	12.69	9.05	10.61	7.41
Lung cancer	14.37	15.45	14.38	17.17	17.29	18.35	22.08	21.45
Maternal mortality	1.85	1.22	0.66	0.32	0.24	0.18	0.15	0.12
Tuberculosis	12.88	7.16	2.72	2.15	1.07	0.79	0.54	0.41
Ulcers	3.57	1.83	1.92	0.84	0.76	0.68	0.93	0.58
Uterine cancer	9.38	6.88	4.98	2.85	1.65	0.99	1.57	2.08
**Urban3**								
**Cause**	**Year**							
	**1971–1975**	**1976–1980**	**1981–1985**	**1986–1990**	**1991–1995**	**1996–2000**	**2001–2005**	**2006–2008**
All causes	284.53	256.46	226.84	202.34	183.85	171.12	150.82	138.35
Appendicitis	0.35	0.09	0.11	0.05	0.07	0.04	0.02	0.04
Asthma	1.11	0.87	0.89	0.63	0.48	0.40	0.36	0.20
Breast cancer	5.73	6.39	7.34	9.21	11.90	14.51	16.85	17.74
Cervical cancer	6.03	6.74	7.07	7.05	8.17	7.96	6.26	4.99
Gallbladder disease	1.03	0.86	0.56	0.25	0.30	0.18	0.26	0.14
Hernia	0.00	0.03	0.01	0.00	0.01	0.00	0.00	0.00
Hodgkin’s disease	0.17	0.14	0.05	0.10	0.04	0.05	0.04	0.08
Hypertension/CVD *	108.24	115.41	103.88	74.53	52.85	35.56	29.89	23.18
Injuries	36.80	42.82	43.83	46.07	41.64	37.73	29.05	25.51
Ischemic heart disease	10.44	11.39	11.97	11.57	12.54	9.72	9.36	8.57
Lung cancer	10.41	11.66	14.35	14.95	16.20	17.75	19.10	22.04
Maternal mortality	2.36	1.33	0.74	0.40	0.27	0.31	0.12	0.22
Tuberculosis	14.37	7.16	3.83	1.88	1.41	0.96	0.52	0.21
Ulcers	5.28	2.84	1.96	1.07	1.27	0.66	0.72	0.60
Uterine cancer	8.56	7.17	6.02	3.63	1.68	1.13	1.45	1.36

Note: * Cerebrovascular diseases.

**Table 2:** Standardized mortality ratio (SMR per 100,000 people), males.

Urban1														
Cause	Year													
	1971–1975	1976–1980	1981–1985	1986–1990	1991–1995	1996–2000	2001–2005	2006–2008
All causes	369.79	351.70	316.12	293.39	284.04	270.44	290.91	268.50
Appendicitis	0.22	0.16	0.10	0.08	0.04	0.03	0.02	0.05
Asthma	0.64	0.63	0.43	0.43	0.53	0.40	0.46	0.33
Gallbladder disease	0.58	0.49	0.36	0.26	0.24	0.24	0.28	0.19
Hernia	0.05	0.06	0.03	0.01	0.01	0.03	0.01	0.01
Hodgkin’s disease	0.39	0.20	0.17	0.05	0.05	0.07	0.10	0.06
Hypertension/CVD *	152.56	149.73	129.88	99.58	79.24	63.66	56.91	49.98
Injuries	72.59	77.04	73.56	71.86	64.00	54.01	50.79	44.95
Ischemic heart disease	22.15	24.99	24.18	27.24	33.22	30.02	34.22	28.87
Lung cancer	26.35	30.23	32.46	32.61	30.74	31.41	37.45	37.11
Tuberculosis	21.68	12.86	7.15	4.57	3.44	2.40	1.66	0.96
Ulcers	7.66	3.84	3.30	2.16	2.16	2.14	2.06	1.39
**Urban2**														
**Cause**	**Year**													
	**1971–1975**	**1976–1980**	**1981–1985**	**1986–1990**	**1991–1995**	**1996–2000**	**2001–2005**	**2006–2008**
All causes	424.88	405.55	334.42	346.80	326.00	310.66	326.74	308.83
Appendicitis	0.14	0.14	0.11	0.06	0.05	0.05	0.05	0.01
Asthma	1.37	0.66	0.60	0.57	0.66	0.39	0.53	0.50
Gallbladder disease	0.49	0.58	0.36	0.21	0.34	0.32	0.29	0.29
Hernia	0.06	0.05	0.04	0.01	0.03	0.00	0.06	0.02
Hodgkin’s disease	0.25	0.30	0.18	0.18	0.10	0.10	0.11	0.05
Hypertension/CVD *	165.09	167.70	128.09	107.90	91.24	71.81	70.16	64.62
Injuries	92.14	103.93	92.54	104.01	89.05	75.09	67.64	60.01
Ischemic heart disease	20.96	22.96	22.63	29.24	33.58	29.64	36.03	31.37
Lung cancer	20.57	26.97	28.60	29.58	33.71	32.12	38.02	39.57
Tuberculosis	26.77	16.13	7.57	5.65	3.93	2.73	2.22	1.32
Ulcers	10.79	6.78	4.31	2.77	2.65	2.56	1.96	1.53
**Urban3**														
**Cause**	**Year**													
	**1971–1975**	**1976–1980**	**1981–1985**	**1986–1990**	**1991–1995**	**1996–2000**	**2001–2005**	**2006–2008**
All causes	451.97	438.00	401.12	381.26	375.94	358.38	357.20	347.33
Appendicitis	0.33	0.24	0.17	0.06	0.04	0.03	0.01	0.01
Asthma	1.40	0.72	0.73	0.75	0.60	0.59	0.63	0.42
Gallbladder disease	0.66	0.76	0.54	0.35	0.46	0.33	0.30	0.25
Hernia	0.13	0.06	0.03	0.03	0.08	0.00	0.01	0.03
Hodgkin’s disease	0.19	0.31	0.21	0.08	0.09	0.14	0.12	0.08
Hypertension/CVD *	148.89	157.25	136.35	105.05	91.42	68.61	70.73	62.89
Injuries	101.15	118.15	123.64	128.42	119.35	103.52	86.43	77.27
Ischemic heart disease	16.57	19.28	22.60	26.29	30.79	25.86	31.06	31.36
Lung cancer	19.01	22.98	29.31	29.31	32.09	32.60	37.73	40.98
Tuberculosis	33.67	19.56	11.56	6.66	4.72	3.41	2.42	1.08
Ulcers	16.86	8.45	5.66	3.94	3.48	3.24	2.45	2.05
**Urban4**														
**Cause**	**Year**													
	**1971–1975**	**1976–1980**	**1981–1985**	**1986–1990**	**1991–1995**	**1996–2000**	**2001–2005**	**2006–2008**
All causes	488.65	484.97	459.85	442.21	446.65	436.33	427.62	420.17
Appendicitis	0.52	0.16	0.25	0.08	0.00	0.00	0.05	0.00
Asthma	1.89	1.08	1.13	0.87	1.08	0.86	0.69	0.89
Gallbladder disease	1.12	0.76	0.61	0.45	0.39	0.46	0.41	0.45
Hernia	0.13	0.03	0.08	0.00	0.01	0.01	0.00	0.00
Hodgkin’s disease	0.25	0.33	0.17	0.05	0.08	0.11	0.24	0.15
Hypertension/CVD *	139.43	155.49	140.57	108.75	93.57	79.40	75.31	72.48
Injuries	105.27	127.34	137.00	151.32	144.48	130.25	105.60	90.73
Ischemic heart disease	13.42	17.55	21.76	25.97	31.99	30.23	34.34	33.80
Lung cancer	19.09	21.32	29.68	34.37	33.97	39.17	42.62	43.80
Tuberculosis	44.01	27.25	16.60	10.18	7.88	6.64	4.29	2.33
Ulcers	20.72	11.54	7.82	5.39	5.32	4.31	2.99	2.80
**Urban5**														
**Cause**	**Year**													
	**1971–1975**	**1976–1980**	**1981–1985**	**1986–1990**	**1991–1995**	**1996–2000**	**2001–2005**	**2006–2008**
All causes	303.42	279.22	245.35	220.13	201.82	195.47	174.69	159.22
Appendicitis	0.31	0.09	0.18	0.06	0.02	0.02	0.06	0.04
Asthma	1.31	0.92	0.94	0.66	0.76	0.59	0.43	0.48
Breast cancer	5.22	5.68	5.93	8.08	10.34	12.39	14.79	15.93
Cervical cancer	5.51	7.23	6.94	6.59	8.31	7.70	7.34	4.95
Gallbladder disease	0.95	0.84	0.51	0.38	0.35	0.32	0.12	0.18
Hernia	0.04	0.05	0.00	0.00	0.00	0.00	0.00	0.00
Hodgkin’s disease	0.06	0.17	0.10	0.02	0.02	0.05	0.10	0.03
Hypertension/CVD *	92.99	100.31	92.48	74.21	52.29	40.71	33.19	28.40
Injuries	38.80	45.44	49.80	52.62	48.34	47.66	35.89	31.33
Ischemic heart disease	7.52	10.32	11.75	13.44	14.56	10.90	11.23	10.60
Lung cancer	10.28	11.86	14.76	15.36	16.26	21.04	20.44	20.24
Maternal mortality	3.84	2.48	1.17	0.40	0.30	0.35	0.21	0.09
Tuberculosis	19.39	10.52	5.95	3.33	2.06	1.92	1.27	0.42
Ulcers	6.82	3.94	2.04	1.20	0.92	0.73	0.61	0.76
Uterine cancer	8.30	9.51	6.43	3.39	1.74	0.79	0.91	1.38

* Cerebrovascular diseases.

## 3. Results and Discussion

### 3.1. Primary Results

#### 3.1.1. All-cause Mortality

Three results stand out when looking at the temporal trend of ASMR disaggregated by urbanization level. First, for both sexes, there is a monotonic relationship between urbanization and ASMR, with the least urban areas demonstrating the highest all-cause ASMR and the most urbanized areas showing the lowest all-cause ASMR. Secondly, these differences in mortality rates persisted over the 38-year study period even as they fell in all areas of Taiwan. Third, ASMR dropped more dramatically for women in Taiwan relative to men, demonstrating a gender-based disparity in death rates.

#### 3.1.2. Avoidable Mortality and Urbanization

Several causes of death had lower ASMR in more urbanized areas than less urbanized areas initially, but the gap converged by the end of the study period. These include tuberculosis (men and women), ulcers (men and women), gallbladder disease (women only), maternal mortality, and to a lesser extent, appendicitis. 

Several causes of death had ASMRs that remained higher in less urbanized areas throughout the entire study period. Deaths from asthma (both genders) injuries (primarily traffic accidents), and gallbladder disease (men only) continued to be lower in more urbanized jurisdictions throughout the 38-year period. Although the differences in ASMR in asthma and gallbladder (for men) between areas of different urbanization levels shrank somewhat from 1970 to 2008, the urban-rural disparities remained visible even by 2006–2008. Injury ASMR followed an inverted U shape for both women and men from 1971 to 2008 in less urbanized areas, and generally fell in the more urbanized areas.

Several ASMRs were initially higher in urban areas, but were overtaken by those in less urban areas by 2006–2008. These include cervical cancer for women, hypertension, ischemic heart disease, and lung cancer. For most of these causes of death, the inversion began in the early 1980s, but for cervical cancer, the switch in ranking between more and less urbanized areas occurred around 1991–1995. 

Breast cancer was the sole cause of death that had consistently higher ASMR in more urbanized areas than in less urbanized parts of Taiwan throughout the study period. Uterine cancer also displayed a unique pattern in temporal trend: It is the only cause of death where urban areas had initially lower ASMR, only to overtake more rural areas by the end of the study period. It is also the only cause of death to have witnessed an uptick in ASMR in all areas of Taiwan beginning in the mid-1990s. ASMR for asthma also reversed its downward trend, rising from 2001–2005 to 2006–2008 among the most rural areas in Taiwan for both genders. Appendicitis ASMR among men also rose in urban areas from 2001–2005 to 2006–2008.

### 3.2. Discussion

The association between urbanization levels and ASMRs due to avoidable deaths should be evaluated in light of Taiwan’s history. In just under fifty years, Taiwan grew from an underdeveloped agrarian island to a leading exporter of high-technology products with the 19th largest economy in the World [32]. Today, Taiwan possesses a vibrant modern economy that holds the World’s sixth-largest foreign exchange reserves ($411.7 billion as of June 2013) [33].

Health care in Taiwan experienced dramatic improvements in accessibility during the study period, particularly with the introduction of universal health care in 1995 [34], but inequities remain in its distribution. Until 1983, the geographic distribution of physicians was determined entirely by market forces, favoring a concentration of providers in urban areas. Even with mandatory assignment of government-subsidized physicians to rural areas beginning in 1983, however, healthcare resources remain scarce in the many of the least urbanized areas in Taiwan today [35]. 

#### 3.2.1. All-cause Mortality

Given these developments in Taiwan’s economy and healthcare resources, it is not surprising to find two consistent patterns emerge in all-cause ASMRs in the study period. First, ASMRs fell in all parts of Taiwan regardless of the level of urbanization. Further, the temporal trends in ASMRs kept a strict ranking order, such that at all time intervals during the study period, areas with the lowest urbanization level (level 4) had the highest all-cause ASMR, followed by levels 3, 2, and 1.

#### 3.2.2 Injuries

ASMRs related to injuries demonstrated monotonically increasing death rates with lower urbanization, perhaps signaling that the greater access to healthcare resources may offset a greater likelihood of harm from accidental injuries in crowded environments [36]. Moreover, higher population densities in urban areas may also contribute to more frequent, but less severe accidents. Of particular interest is the inverted U shape of the temporal trends in injury-related ASMRs, especially for the least urbanized areas. The timing of this reversal coincided with Taiwan’s introduction of helmet laws in 1997, suggesting that this safety legislation was effective in reducing the burden of injury-related death rates [37].

#### 3.2.3. Gender-specific ASMRs

Many of the risk factors for several gender-specific ASMRs appear more intimately linked to urban lifestyles. For cervical cancer, these include earlier sexual activity, multiple partners, and human papillomavirus (HPV) infection. For breast cancer, the risk factors are many, but those related to urban lifestyle choices include late or no pregnancies, not breastfeeding, alcohol consumption, obesity and physical inactivity. Yet the ASMRs from these two gender-specific diseases displayed different temporal patterns based on urbanization levels during the study period.

Cervical cancer ASMRs in less urban areas, which began lower, eventually caught up with and overtook those in urban areas, leading to a reversal of rankings in ASMR by approximately 1991–1995. This reversal coincided with screening initiatives beginning in the 1990s. In 1991, the National Labor Insurance began covering Pap smears for all women in the private and public sectors [38], who were likely urban residents. In 1995, Taiwan launched its National Health Insurance program [34], and began paying for annual Pap tests for women aged 30 and older. Previous research has established the effectiveness of cancer screening programs in reducing cervical cancer mortality [39]. Our results suggest that the most urbanized area may have benefited from the ease of access to health care.

The medical literature also shows a mortality benefit for breast cancer screening [40]. Mortality in the West has been decreasing despite rising incidence. Yet both incidence and mortality for breast cancer are rising in Asia [41]. Breast cancer screening may be underutilized in Taiwan because of a perception of low incidence and difficulty of assessing parenchyma mammographically in Asian women [42]. This low uptake may help explain why breast cancer mortality is one of the few causes of death with continued upward growth in Taiwan, and the only one with higher ASMR in urban areas throughout the study period despite easier access to care.

#### 3.2.4. Chronic Illnesses

##### Asthma

Asthma attacks have been shown to be triggered by numerous environmental factors, including low temperature, low humidity, and traffic pollutants [43]. Despite higher pollutions and industrial allergens in urban areas, however, ASMRs due to asthma were higher in the least urbanized towns and villages in Taiwan for both sexes in every period of our sample. These trends suggest the greater access to health care [44] may have helped temper the environmental disadvantages with respect to asthma ASMRs in urbanized settings.

##### Hypertension and Cerebrovascular Diseases

Initially, more urbanized areas had higher ASMRs for these two causes of mortality, but less urban areas subsequently overtook their urbanized counterparts. The inversion point began approximately in the 1981-85 period for both genders [45]. During this period, case-fatality rates for hemorrhagic strokes declined rapidly in Taiwan [46], and the prevalence of (lower fatality) cerebral infarctions increased relative to that of (higher fatality) hemorrhagic strokes. The combination of access to emergency medical care in resource-rich urban areas and improvements in the case-fatality rates of strokes may help explain the inversion in the rankings of hypertension-related ASMRs based on urbanization levels.

##### Ischemic Heart Disease

IHD depends critically on resource-intensive emergency care once it occurs. As Taiwan’s health care infrastructure grew along with its economic development, the increases in hospital and tertiary care centers in urban areas may have contributed to the reversal of post-IHD survival rates between more urbanized and less urbanized areas of Taiwan in the 1980s.

Furthermore, atherosclerosis requires consistent, long-term care and management to prevent the development of ischemia. Despite common adverse health behaviors associated with urban lifestyles, greater accessibility to primary care and/or to emergency medical care may explain the slower growth or reduction in IHD ASMRs in urban relative to more rural settings.

##### Lung Cancer

ASMRs for lung cancer represent one of only three causes of avoidable mortality that rose during the study period. Furthermore, ASMRs for lung cancer either converged between urban and rural areas (for women), or inverted rankings, with less urbanized areas overtaking the more urbanized areas (for men). This trend may reflect a number of issues. 

Globally, tobacco smoking is the primary cause of lung cancer [47,48,49,50,51,52]. The prevalence of cigarette smoking in Taiwan rose dramatically since the 1950s [53]. When disaggregated by the level of educational attainment, smoking prevalence reached as high as 73.5% among men aged 18 to 39 who did not finish high school [54]. The first large-scale survey on tobacco smoking conducted by researchers in Taiwan found that rural counties such as Ilan, Taichung, Taoyuan and Miaoli had the highest prevalence of smokers in Taiwan. The same study also confirmed that rural counties as a whole had more smokers than urban areas, and that even geographically proximate city-county pairs invariably had higher prevalence in the county than in the metropolitan areas [55]. The rising prevalence of smokers in rural Taiwan likely explains why ASMRs for lung cancer in rural areas have overtaken those of the more urbanized areas from 1971 to 2008.

Indeed, it is estimated that unless Taiwan is successful in reducing smoking rates by more than 4% a year, mortality attributable to smoking will continue to increase in Taiwan [56]. Taiwan’s enactment of the Tobacco Hazards Prevention Act in 2009 represents an encouraging trend, and the government plans to tackle tobacco control even more aggressively in the coming years. Rural areas should be a top priority given the trend in lung cancer ASMR.

### 3.3. Limitations

There are several limitations to this study. First, ICD-9-CM assignment errors in cause-of-death determinations may exist even though Taiwan’s household death registry is considered excellent [26]. However, there would have had to be systematic errors in assignment between areas of different urbanization levels and between different years for the trends to be misinterpreted in this study. 

Moreover, this study is based on a classification of urbanization determined in one single year, and does not take into consideration changes in urbanization levels over the study period. However, to the extent that the difference in urbanization levels between jurisdictions remain relatively stable across the years, this study may still capture differences in mortalities between more and less urbanized areas even as most jurisdictions urbanized over the 38-year period in Taiwan. 

## 4. Conclusions

With a few rare and notable exceptions, more urbanized areas in Taiwan had lower ASMRs for both avoidable deaths as well as all-cause mortality. ASMRs were lower historically in more urbanized areas for all-cause mortality, and for appendicitis, asthma, injuries, and maternal mortality. ASMRs in more urbanized areas began higher, but eventually fell to rates lower than those in less urbanized areas for hypertension/ cerebrovascular diseases, lung cancer among men, and cervical cancer among women. Many of the lower ASMRs in more urbanized areas were achieved despite less favorable environmental or epidemiological factors in urban areas. A possible explanation for the lower ASMRs in urbanized areas may be the richer healthcare resources in Taiwan’s cities and towns.

The only cause of death to exhibit higher ASMRs in more urbanized areas throughout the study period is death due to breast cancer. Moreover, among avoidable deaths, only three ASMRs rose in our 38-year study period: breast cancer (for women only) and lung cancer for all levels of urbanization, and ischemic heart disease (for all levels of urbanization among men, and for the most rural areas among women). 

These findings imply that policymakers should consider the urban-rural divide evident in almost all causes of mortality in Taiwan when allocating scarce healthcare resources, and make additional investments to combat the three causes of death that rose from 1971 to 2008. In particular, Taiwanese policymakers should consider further encouraging breast cancer screening, and aggressively pursuing anti-tobacco initiatives, especially in rural areas. Future studies should investigate the reasons for greater breast cancer ASMRs in urban areas despite richer healthcare resources in such areas in order to craft a tailored policy response to the growing mortality rates from breast cancer. In addition, further studies should explicitly study the link between healthcare resources and mortality rates in Taiwan for numerically important causes of deaths.
